# Fecal stress, nutrition and reproductive hormones for monitoring environmental impacts on tigers (*Panthera tigris*)

**DOI:** 10.1093/conphys/coz091

**Published:** 2020-01-12

**Authors:** Samrat Mondol, Rebecca K Booth, Samuel K Wasser

**Affiliations:** 1 Center for Conservation Biology, Department of Biology, University of Washington, Box 351800, Seattle Washington 98195-1800; 2 Wildlife Institute of India, Chandrabani, Dehradun 248001, India

**Keywords:** Adrenal, conservation physiology, stress-nutrition interplay, thyroid and reproductive hormones assay validation, tigers

## Abstract

Non-invasive stress and nutritional hormone analysis in relation to ecological and other biological indices have tremendous potential to address environmental disturbance impacts on wildlife health. To this end, we examined the relation between glucocorticoid (GC) and thyroid (T3) hormone indices of disturbance and nutritional stress in response to ACTH and TSH challenges in captive tigers, as well as how reproductive hormones vary by sex and reproductive condition. Glucocorticoid, thyroid, progesterone and androgen assays conducted on high-performance liquid chromatography separated fractions of biologically relevant fecal extracts revealed high cross-reactivity of these assays for their respective biologically relevant fecal hormone metabolites. Both adrenal and thyroid hormone metabolites were elevated in response to ACTH and TSH challenges. However, the adrenal and thyroid hormone responses to ACTH challenge were concurrent, whereas the adrenal response to TSH challenge was delayed relative to thyroid hormone elevation in both males and females. The concurrently elevated T3 in response to ACTH may serve to raise metabolic rate to maximize use of GC-mobilized glucose, whereas the relatively delayed GC rise following TSH challenge may be a response to glucose depletion due to increased metabolic rate associated with elevated T3. Progesterone, testosterone and androstenedione hormone metabolites were significantly elevated during gestation compared to lactation in a female monitored from conception through early lactation. Results suggest that the glucocorticoid, thyroid and reproductive hormone assays we tested can accurately measure the stress, nutrition and reproductive response from tiger feces, providing useful non-invasive tools to assess physiological responses to environmental stressors and their reproductive consequences in the wild.

## Teaser

Glucocorticoid, thyroid, progesterone and androgen hormones are shown to reliably reflect biological activity of adrenal, thyroid and gonadal activation in captive tigers. Combined ACTH and TSH challenges further suggest how adrenal and thyroid hormone secretion complement one another to coordinate the physiological response to nutritional stress.

## Introduction

Fecal hormone analysis has become an increasingly valuable method for studying physiology of free-ranging animals. The power of this approach stems from the accessibility of fecal samples and the variety of reproductive, stress and nutrition hormones that can be measured in fecal samples (Wasser *et al.*, 1996, 2004, 2010, 2011, 2017; Creel *et al.*, 2002; Hunt and Wasser, 2003; Keay *et al.*, 2006; Gobush *et al.*, 2008; Hayward *et al.*, 2011). Simultaneous measurement of other biological indices in these same samples further adds to the utility of this approach, including DNA from the host, prey, microbiome, toxins and ecological measurements associated with temporal and spatial variation at the time and place of sample collection (Wasser *et al.*, 2004, 2011, 2017; Lundin *et al.*, 2016). The growing impacts that humans continue to have on wildlife through habitat destruction and other environmental insults further add to the importance of these methods. For example, temporal and spatial sample collection in relation to changes in environmental pressures can be used to partition impacts of psychological vs. nutritional stress in relation to human disturbance (Wasser *et al.*, 2017) as well as the effect they have on reproductive success such as pregnancy occurrence, health and failure (Gobush *et al.*, 2008; Strasser and Heath, 2013).

The ways in which hormones such as glucocorticoid (GC) and bioactive thyroid hormone (triiodothyronine, T3) complement one another adds yet another powerful tool to this arsenal (Wasser *et al.* 2011, 2017). GCs act to mobilize glucose, providing emergency energy to respond to immediate environmental pressures such as a predator attack or lack of food (Sapolsky *et al.*, 2000). T3 generally acts more slowly, raising or lowering metabolism in response to environmental demands such as acute or chronic nutritional stress (Wasser *et al.*, 2010). However, the combined actions of these two hormones in regulating physiological responses to the environment under different contexts can be equally revealing. If glucose is required to address an immediate need such as escape from a predator or finding food, elevations of GC to mobilize glucose might be most functional if associated with elevated T3, so the resultant elevated metabolism can make best use of the increased availability of glucose. On the other hand, as food stress becomes more persistent, chronically elevated GC might deplete internal reserves, making it important to reduce T3 and associated metabolism to prevent the body from using all its remaining reserves (Wasser *et al.*, 2017). Persistently elevated T3 to serve any number of physiological functions might also result in depleted glucose, creating a need for increased GCs in compensation. This study validates a number of fecal hormone measurements in captive tigers for eventual application to the wild, simultaneously examining the relation between GC and T3 secretion in response to both adrenocorticotrophic hormone (ACTH) and thyroid-stimulating hormone (TSH) challenges.

Tigers (*Panthera tigris*) exemplify globally endangered large conservation species with only about 3500 individuals remaining in the wild. Growing anthropogenic pressures in the form of habitat and prey depletion, poaching and human–animal conflict are currently major concerns for their future existence (Sanderson *et al.*, 2006; Morrison *et al.*, 2007). However, the physiological and reproductive impact of environmental disturbances and their importance in conservation efforts has received little research attention, particularly in the wild. Long-term survival for these carnivores will also need to address the human-induced problems since most of the remaining tiger habitats are disjunct and fragmented (Ranganathan *et al.*, 2008). To date, significant conservation efforts have been made to increase local population size and connectivity among populations (Walston *et al.*, 2010), but few attempts have been made to understand the underlying impacts from human population pressure, habitat fragmentation and limited food resources on the health and reproductive functions of these large felines (for example, see stress measures on tigers in Bhattacharjee *et al.*, 2015; Naidenko *et al.*, 2019; Tyagi *et al.*, 2019). Non-invasive physiological measures of psychological and nutritional stress as well as their reproductive consequences may prove useful here by providing indices to bridge the intervals between multiple environmental disturbances and their population responses. Such information is vital to guide efforts aimed at curbing population declines. Species-specific variations in hormone metabolites excreted in feces (Wasser *et al.*, 1994; Palme *et al.*, 1996; Schwarzenberger *et al.*, 1996) make it important to validate fecal assays across hormones and species before large-scale field use. While several papers have been published on tiger adrenal and gonadal hormones (Brown *et al.*, 1994; Graham *et al.*, 1995; Putranto, 2011; Umapathy *et al.*, 2013; Bhattacharjee *et al.*, 2015; Naidenko *et al.*, 2019; Tyagi *et al.*, 2019), the antibodies used to measure each hormone as well as in the extent of their validations vary markedly. These differences make comparisons between studies difficult because different antibodies have varied affinities for the metabolites of the parent hormones they were raised against and thus can only be compared within vs. between studies. High-performance liquid chromatography (HPLC) results could be compared but only if the two studies used identical solvents and elution profiles. The antibodies we examined in our study were chosen for their broad applicability across species (Wasser *et al.*, 1994, 2000, 2010), including felids (Brown *et al.*, 1994), and are extensively validated in this study using parallelism, accuracy and HPLC to show that these assays accurately measure the chosen hormones across their ranges of concentration. Most importantly, concurrent ACTH and TSH challenge studies were conducted to show how these two hormones collectively reflect biological function.

The following fecal hormones were validated for tigers in this study.

(1) Glucocorticoid hormones: Cortisol is the primary glucocorticoid secreted in most large mammals while corticosterone is primary in birds and several small mammals. However, Wasser *et al.* (2000) showed that cortisol is rapidly metabolized in most large mammals and thus best measured in fecal samples by corticosterone assays. Despite having a myriad of effects upon multiple systems, the primary function of glucocorticoids is to facilitate glucose release in response to an acute stressor. Glucocorticoids have been used to study a range of stress-related physiological phenomena in a range of animals, e.g. population abundance trajectory (Boonstra *et al.*, 1998), predator pressure (Boonstra *et al.*, 1998; Clinchy *et al.*, 2004), social structure and relationships (Pride, 2005; Raouf *et al.*, 2006; Rubenstein, 2007; Gobush *et al.*, 2008), immune state or infection status (Boonstra *et al.*, 1998; Hanley and Stamps, 2002; Raouf *et al.*, 2006), reproductive state/hormone titers (Boonstra *et al.*, 1998; Hunt *et al.*, 2006), body condition/injury state (Romero *et al.*, 2000; Wingfield and Romero, 2001; Hunt *et al.*, 2006), environmental conditions (Smith *et al.*, 1994; Astheimer *et al.*, 1995; Romero *et al.*, 2000; Schoech *et al.*, 2009, Busch and Hayward, 2009) and nutritional stress (Wasser *et al.*, 2011, 2017).
(2) Thyroid hormone: Thyroid hormone is known to have important influences on metabolism, blood pressure, body temperature regulation and nutritional physiology (Oppenheimer, 1999; Silva, 2003, 2006; Wasser *et al.*, 2010, 2011, 2017). Thyroid hormones (both thyroxine, T4 and triiodothyronine, T3) are particularly responsive to nutritional fluctuations, by lowering metabolism during nutritional emergency, but appear relatively unresponsive to psychological stress (Kitaysky *et al.*, 2005; Wasser *et al.*, 2010). Due to these distinctive functions Thyroid hormones are particularly useful to characterize nutritional stress in disturbance biology (Wasser *et al.*, 2011, 2017; Behringer *et al.*, 2018).
(3) Aldosterone: Aldosterone is one of the major mineralocorticoids in most species, its primary role being physiological regulation of electrolytes (sodium retention and potassium retention) and fluids (blood pressure) and coping of stress (Weeks and Kirkpatrick, 1976). Several studies have also documented aldosterone secretion in response to ACTH during time of stress (Houser *et al.*, 2011). ACTH improves blood flow to organs and muscles and facilitates glucocorticoid circulation throughout the body (Houser *et al.*, 2011). It also inhibits excess energy utilization from fat stores, thereby helping longer-term persistence in times of fluctuating energy sources.
(4) Progesterone: Progesterone provides an important index of ovulation and pregnancy in mammals (Monfort *et al.*, 1993; Wasser, 1996; Velloso *et al.*, 1998; Wasser *et al.*, 1996,^1998^, ^2011^, ^2017^; Wasser and Hunt 2005).
(5) Testosterone: Testosterone and other metabolites (e.g. Androstenedione and DHT), commonly called androgens, are used to investigate reproductive state, sex, behaviour and social rank in many mammals (Creel *et al.*, 1997; Barrett *et al.*, 2002; Dloniak *et al.*, 2004; Wasser and Hunt 2005; Ayres *et al.* 2012) and pregnancy occurrence (Wasser *et al.*, 2017). In this study, we have validated both testosterone and androstenedione (henceforth A4) metabolites from tiger feces.

We used four approaches to validate the above hormone assays on tigers. (i) Parallelism and accuracy tests were used to respectively demonstrate that our assays were reliably measuring hormone metabolites across their ranges of concentration and that nothing in the fecal samples is interfering with hormone measurement (Christopolous and Diamandis 1996). (ii) HPLC was used to determine the predominance of fecal hormone metabolites and validate their affinities to respective assays. (iii) Pharmacological challenges with ACTH and TSH were conducted on captive male and female tigers to assess biological activity of the adrenal hormone metabolites being measured as well as the lag time between their secretion and excretion in fecal samples. (iv) We followed the entire reproductive cycle (gestation, lactation) of a female tiger and collected feces to examine differences in fecal progesterone, aldosterone, testosterone and androstenedione metabolites from prior to conception through gestation and early lactations in a single captive female.

## Materials and methods

### Study subjects

This study was conducted on two pairs of male and female tigers, one pair housed at Point Defiance Zoo and Aquarium (PDZ), Tacoma, WA and the other at Henry Doorly Zoo and Aquarium (HDZ), Omaha, NE. The pair at PDZ were ages 5 (M) and 11 (F) years, and the pair in HDZ were ages 6 (M) and 7 (F) years. All hormone challenges and sample collections were conducted in compliance with the University of Washington IACUC and welfare committee permits from respective zoos.

### Sample collections

Samples were collected pre- and post-hormone challenges in both zoos. The ACTH challenges were conducted during 1–9 June 2012 (HDZ—ACTH) and 10–23 December 2012 (PDZ—ACTH). The TSH challenges were conducted 27 November–10 December 2012 (PDZ—TSH). All fresh fecal samples were collected in the morning from the enclosures where the animals were separately housed during the entire study. All samples were placed in sealed sterile zip-lock bags using sterile gloves and labelled by animal name, sample number and date. All samples were stored immediately in a −20°C freezer at the zoo, and subsequently shipped to the University of Washington on dry ice, where they were kept at −20°C until processing.

**Table 1:** Details of the hormone assays conducted in this study

**Hormone**	**Assay method**	**Dilution**	**Slope (R** ^**2**^ **)**	**Inter-assay CV**	**Intra-assay CV**	**Cross-reactivity**
Corticosterone	RIA	1:200	1.09 (0.99)	3	11	Corticosterone 100%, desoxycorticosterone 0.34%, testosterone 0.25%, cortisol 0.05%, aldosterone 0.03%, progesterone 0.02%, androstenedione 0.01%, 5A-dihydrotestosterone 0.01% and <0.01% for all other tested steroids
Triiodothyronine (T3)	RIA	1:30	1.12 (0.99)	2	11	*L*-triiodothyronine 100%, *L*-thyroxine 0.18%, 3,5-diiodothyronine 0.44%, 3,3′5′*L*-triiodothyronine (r-T3) 0.01% and <0.01% for 3,5-diiodotyrosine, phenylbutazone, sodium salicylate, diphenylhydanation and dicumerol
Thyroxine (T4)	RIA	1:10	0.94 (0.99)	1	-	*L*-thyroxine 100%, *D*-thyroxin 64%, tetraiodothyroacetic acid 1.4%, triiodo-*L*-thyronine 2%, triiodothyroacetic acid 2% and nondetectable levels of triiodo-*D*-thyronine, monoidotyrosine, diiodo-*L*-tyrosine, methimazole, 5,5′ diphenylhydantoin, phenylbutazone and 6-*n*-propyl-2-thiouracil
Aldosterone	RIA	1:75	1.10 (0.99)	3	-	Aldosterone 100%, progesterone 0.007%, Corticosterone 0.002%, 18-OH-Corticosterone 0.033%, 11-Deoxycorticosterone 0.006, Cortisone 0.0003%, 11-Deoxycortisol 0.0004%, Dexamethasone 0.00005%, DHEA 0.0005%, Spironolactone 0.06%
Progesterone	RIA	1:60	0.99 (0.99)	2	8	Progesterone 100%, 17b-OH-progesterone 15%, pregnenolone 13%, 20a-hydroxyprogesterone and estrone 1% and <1% for all other tested steroids
	EIA	1:360	1.01 (0.99)	3	4	Progesterone 100%, 3a-hydroxy-progesterone 188%, 3b-hydroxy-progesterone 172%, 11a-hydroxy-progesterone 147%, 11b-hydroxy-progesterone 2.7%, 5a-dihydroprogesterone 7%, pregnenolone 5.9%, corticosterone <0.1%, androstenedione <0.1%
Testosterone	RIA	1:30	0.96 (0.99)	3	10	Testosterone 100%, dihydrotestosterone 69%, 3b-androstanediol 22%, 3a-androstanediol 14%, androst-4-ene-3 and 17-dione 1%, 17b-estradiol 0.3% and <0.01% for all other tested steroids
	EIA	1:150	1.07 (0.99)	2	5	Testosterone 100%, 5a-dihydrotestosterone 56.8%, androstenedione 0.27%, androsterone 0.04%, DHEA 0.04%, Cholesterol 0.03%, 17b-estradiol 0.02%, progesterone <0.02%, pregnenolone <0.02%, hydrocortisone <0.02%, cholic acid and derivatives <0.02%
Androstenedione (A4)	RIA	1.300	1.12 (0.99)	2	-	Androstenedione 100%, DHEA-S 4.4%, DHEA 3.5%, estrone 1.79%, testosterone 0.64%, Progesterone 0.07%, 17B-estradiol 0.02% and <0.01% for aldosterone, cholesterol, corticosterone, cortisol, dihydrotestosterone, desoxycorticosterone, 11-desoxycortisol, estriol, 17A-hydroxyprogesterone, pregnenolone, pregnenolone sulphate and 17A-hydroxypregnenolone

Fecal samples were collected three times per week from May to September 2012 over the entire duration of pregnancy from the PDZ female, spanning the day of mating through the first 2 weeks of lactation. Progesterone rises post-ovulation and becomes increasingly elevated post-implantation in eutherian mammals, emulating a natural challenge for reproductive hormones.

### Hormone challenges

ACTH challenges were conducted on the male and female tigers housed at Point Defiance and Henry Doorly Zoos, whereas TSH challenges were conducted only on the male and female tigers on the PDZ. During all challenges, all fresh fecal samples were collected 0–3 days prior to and then 5–7 days following injection. Due to excretion lag time (up to 30 hours in mammals), samples collected at time 0 (injection time) were considered as pre-injection samples. We used a dose of 4-mg/kg body weight ACTH (Sigma-Aldrich; Crystalline form, reconstituted with saline) for ACTH challenges in all four tigers (two from each zoo), delivered in the morning in a single intra-muscular injection.

The TSH challenge used a newly developed TSH analog, TR1402 (Trophogen Inc., MD), on the PDZ tiger pairs. TR1402 is a pure recombinant human TSH analog that is 10–50 times more potent than other available TSH analogs (Reinfelder *et al.*, 2011). The animals received a single injection of 1.5 mg TR1402 to produce a sustained adrenal activation. This dose was calculated following discussions with three independent thyroid experts (B. Anawalt, M. Soules and B. Weintraub, personal communication), considering the higher potency of this new analog, the standard dose for TSH and the potential health hazards to the test subjects. The male and female were housed separately during hormone challenge studies.

### Hormone extraction

To control for moisture and diet variation, all frozen fecal samples were lyophilized prior to hormone extractions in a Labconco FreeZone Freeze Dry System at −50°C for a minimum of 96 hours (Wasser *et al.*, 1993). Lyophilized samples were then sifted through a 1-mm stainless steel mesh to pulverize and remove any hair or hard parts, resulting in a homogenized powder. Subsequently, hormone extraction was performed on all samples by pulse-vortexing 0.1 grams of freeze-dried fecal powder in 15 ml 70% ethanol. Each pellet was extracted twice and the two 15 ml extracts pooled, as described in Wasser *et al.* (2010).

### HPLC analyses

Reverse phase HPLC analyses were conducted using multiple fecal samples from the same male and female tiger (PDZ), combined to create a single pool for each sex. Each pool was extracted in triplicate using 0.2 grams of well-mixed lyophilized fecal powder, homogenized in 5 ml of 70% ethanol using the pulse-vortex method previously described. The triplicate extracts from each pool were then combined and extracts were cleaned by passing through a 0.2-μm filter followed by C-18 Bond Elut cartridge (Varian Instruments, Walnut Creek, CA) and eluted with 5 ml of methanol as described in Wasser *et al.* (2010). The eluent was dried under forced air, reconstituted in 500 μl of 100% methanol and stored at −20°C until HPLC injection.

Following purification, each 100 μl fecal extract was spiked with a ^3^H radiolabeled reference standard, injected onto a C-18 reverse phase HPLC column (Varian Instruments) and all fractions collected at a flow rate of 1 ml/min. Fecal glucocorticoids were separated using the following methanol/water gradient solvent system: 20–30% methanol (0–10 min), 30–40% (10–40 min), 40–50% (40–55 min), 50–80% (55–80 min), 80–100% (80–85 min) and 100% (85–120 min) (Wasser *et al.*, 2000). For separation of T3 and T4, a methanol/ammonium acetate (pH 4.0) gradient was used, with methanol increasing linearly from 45–65% over 40 min (Wasser *et al.*, 2010). Testosterone metabolites were separated using an isocratic 35% acetonitrile/65% water gradient over 80 min (modified from Dloniak *et al.*, 2004). To assess retention time of the ^3^H radiolabeled reference standards, 100 ul of each fraction was counted for radioactivity, and the remainder was dried under forced air and resuspended in 500 μl distilled water.

### Hormone assays

Fecal extracts were dried down and resuspended in assay buffer at appropriate dilutions (see below) for each of nine different assays: ^125^I Corticosterone Double Antibody RIA kit (#07-120 103; MP Biomedicals), ^125^I Total T3 coated tube assay kit (#06-B254216; MP Biomedicals), ^125^I Total T4 Coat-A-Count coated tube assay kit (#10381554; Siemens Healthcare Diagnostics, Tarrytown, NY), ^125^I Aldosterone Coat-A-Count coated tube assay kit (#10381343; Siemens Healthcare Diagnostics), in house 3^H^ progesterone RIA assay (Rolland *et al.*, 2005) as well as a progesterone EIA kit, both using the same CL425 antibody (#K025-H5; Arbor Assays, Ann Arbor, MI), in-house ^3^H testosterone RIA assay using antibody #250 (Rolland *et al.*, 2005), a testosterone EIA using antibody R156/7 (#K032-H5; Arbor Assays) and an ^125^I Androstenedione Double Antibody kit (#07109202; MP Biomedicals). The cross-reactivities of each of these assays are listed in Table 1.

### Parallelism and accuracy studies

Parallelism tests for each assay were conducted using serial dilutions of pooled fecal extract from multiple male and female samples to assess reliable quantification of metabolites at different concentrations as well as to find optimal dilutions for final assays (at 50% binding). For all these tests, sigmoid graphs were generated as relative dose vs. percent bound hormones for the pools and the standards. Parallel slopes indicate that the antibody binds well to the fecal metabolites across a range of concentrations.

Accuracy tests were next conducted for each assay to determine any interference during antibody interactions. Hormone standards were spiked with equal volume of diluted fecal extract of known concentration (dilution level close to 50% bound from parallelism test) and assayed alongside standards. Results were graphed as known standard dose vs. apparent dose (spiked standard concentrations minus the added dose from the spike) with a slope of 1 (range from 0.9 to 1.1) indicating that fecal components were not interfering with assay accuracy at the tested dilution. All dilutions used and slopes of accuracy lines can be found in Table 1.

**Figure 1:**
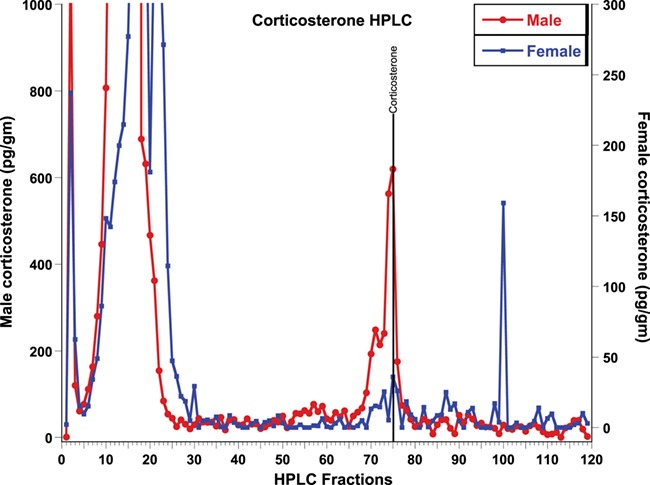
Result of tiger fecal corticosterone HPLC fraction assays for both sexes

### Statistical analyses

Parallelism test results were examined with an F ratio test using the program PRISM to test for differences in slopes. Accuracy results were compared with Student’s *t*-test.

Statistical analyses were conducted in SPSS© version 23 and R statistical software (R Studio, 2012). Pre- and post-challenge fecal hormone metabolite values of all subjects were pooled, and exploratory analyses (scatter plots and Q-Q plots) were conducted to test sample distribution. Since the data did not fit the Gaussian distribution (possibly due to low sample size), we relied on non-parametric statistical tests to quantify pre- and post-challenge endocrine changes within subjects. We used Wilcoxon signed rank test to compare pre- and post-challenge fecal hormone levels within test subjects. We also explored the inter-relationship between T3, T4, cortisol and aldosterone levels post-challenge using Spearman’s rank correlational coefficient to investigate monotonicity between post-challenge hormone levels. We measured fecal progesterone, testosterone, androstenedione and aldosterone in a female tiger at normal, gestation and lactation conditions. Hormone levels between pre-pregnant and gestation, and pre-pregnant and lactation conditions were compared for each hormone using Wilcoxon signed rank test.

## Results

### Parallelism and accuracy

Parallelism and accuracy studies of the targeted hormones indicated that we are reliably measuring all fecal hormone metabolites across their ranges of concentration, with the possible exception of the T and T4 RIAs. Both of those hormone assays showed good parallelism and accuracy, but the undiluted samples barely reached 50% bound for T and never reached 50% for T4. The T EIA, however, worked very well and we recommend use of that antibody for measurement of T. While the T4 antibody may require further investigation at higher hormone concentrations, T3 is far more important to measure due to its significantly higher biological activity relative to its T4 precursor (Wasser *et al.*, 2011) and the T3 assay validated in this study performed very well. Serial dilutions of fecal extracts paralleled respective standard curves in all cases (Supplementary Figs S1–S9) and 50% binding occurred at a 1:10 to 1:360 dilutions, depending on hormones and assay approaches (Table 1). Hormone standards spiked with tiger fecal extracts produced slopes ranging from 0.94 to 1.12 depending on hormones, illustrating that fecal extracts did not interfere with their measurement precisions (Table 1). Thus, with the possible exception of the T and T4 RIAs, all assays validated in this study accurately and reliably measured the respective hormone metabolites across different concentration ranges, without any interference from fecal products. Inter-assay coefficient of variation (CV) ranged from 1 to 3 and intra-assay CV’s ranged from 4 to 11, depending on the assay (Table 1).

### Assay comparison (RIA vs. EIA)

No significant difference was found between EIA and RIA assays for testosterone (mean testosterone EIA = 134.58 ± 15.69 ng/g vs. RIA = 139.32 ± 25.23 ng/g; *P* = 0.43; *r* = 0.9; *P* = 9*10^−12^) or progesterone (mean progesterone EIA = 184.77 ± 113.23 ng/g vs. RIA = 143.02 ± 95.34 ng/g; *P* = 0.39; *r* = 0.99; *P* = 5*10^−8^). As both approaches showed reliable and accurate hormone measurements through parallelism and accuracy studies, we decided to use the EIA approach for both hormones in all subsequent assays due to ease of handling and faster processing time.

**Figure 2:**
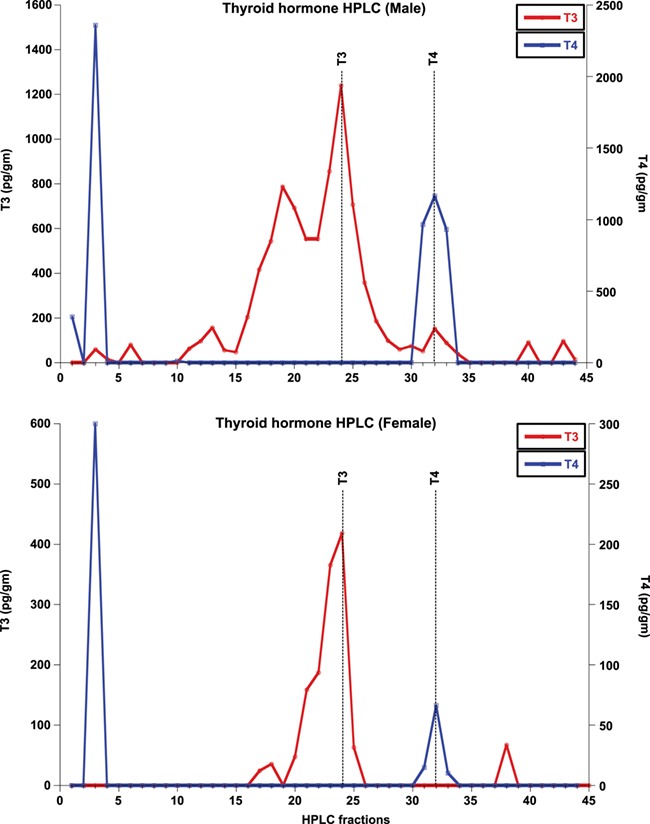
HPLC fraction analyses of tiger fecal thyroid hormone (T3 and T4) in both sexes. The top and bottom panels show the results for male and female tigers, respectively

### HPLC analyses

**Figure 3:**
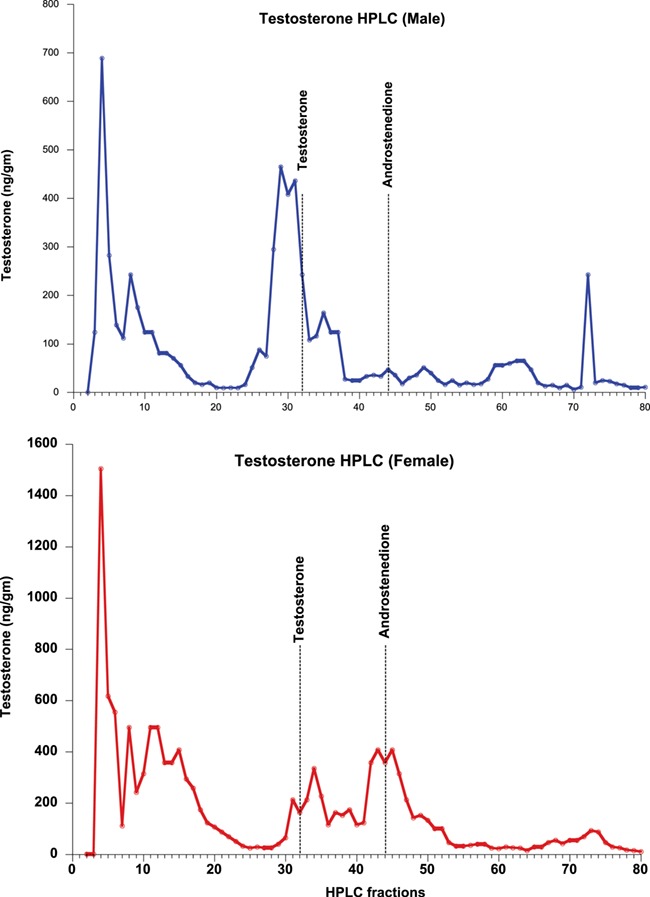
HPLC fraction analyses of tiger fecal androgens (Testosterone and androstenedione) in both sexes. The top and bottom panels show the results for male and female tigers, respectively

**Figure 4:**
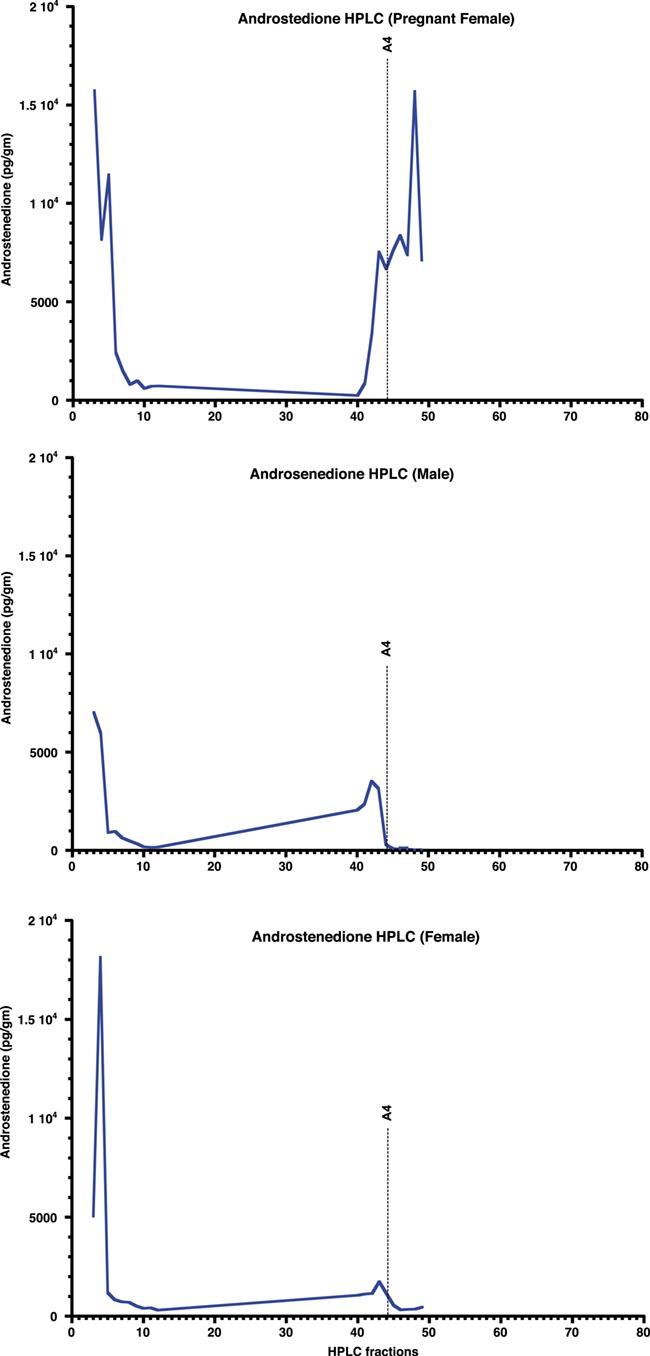
HPLC fraction analyses of tiger fecal androstenedione in both sexes. The top and bottom panels show the results for a pregnant female and male tigers, respectively

HPLC-separated fractions of glucocorticoid, thyroid and androgen metabolites were each tested for immunoreactivity using the assay kits described in the methods. Elution profiles for all three hormones included highly polar fecal metabolites, found in fractions 1–20, depending on the hormone (see Figs 1–4). The corticosterone antibody detected a relatively sharp immunoreactive peak for both sexes eluting in the same fraction as corticosterone (fraction 75), with some cross-reactivity to fractions 71–75 (Fig. 1). The female peaks were smaller than those for the male, suggesting potential sex differences in hormone metabolization (Fig. 1). Both T3 and T4 antibodies detected immunoreactive peaks in fractions 24 and 32 for both sexes, corresponding to where T3 and T4 respectively elute (Fig. 2). Both thyroid hormones were present at higher levels in males than they were in females. HPLC fractions assayed with the testosterone EIA detected peaks eluting consistent with testosterone (fractions 28–32; Fig. 3) and A4 (fractions 42–45; Fig. 4), when compared to standard peaks for respective hormones (fraction 32 for testosterone and fraction 44 for A4). Testosterone was present at higher concentrations in males, whereas A4 was higher in females.

### ACTH and TSH challenges

**Figure 5:**
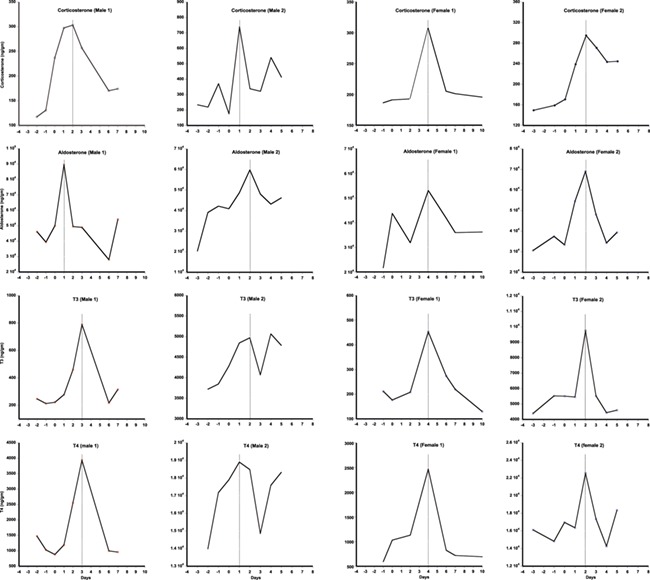
Results of fecal corticosterone, aldosterone, T3 and T4 assays following ACTH challenges on male and female captive tigers (*n* = 2 for each sex). In X-axis 0 represent the day of ACTH challenge

**Figure 6:**
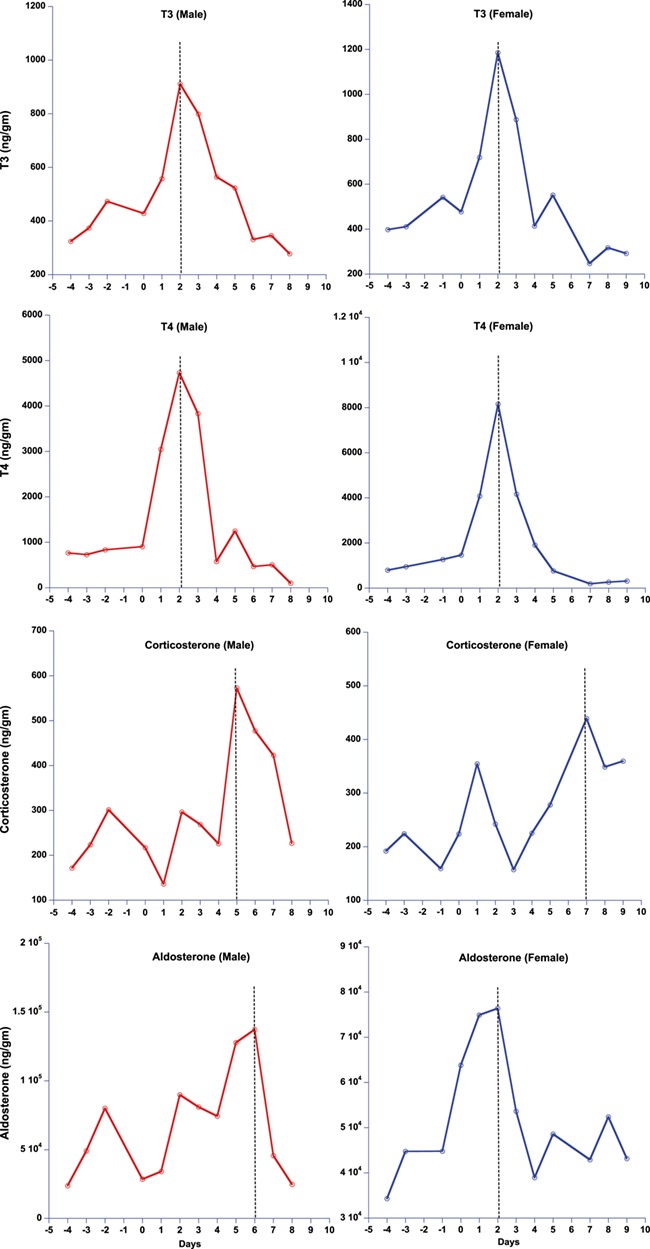
Results of fecal T3, T4, corticosterone and aldosterone assays following TSH challenges on male and female captive tigers (*n* = 1 for each sex). In X-axis 0 represent the day of TSH challenge

All individuals (including both sexes) challenged with ACTH and TSH responded physiologically with an increase in respective fecal hormones (Corticosterone, T3 and T4) from average pre-challenge baseline levels (Figs 5 and 6). The latency to peak GC concentration varied between genders, with males showing an increase between 1–3 days post-challenge and 2–5 days for females (Fig. 5). Average peak fecal corticosterone levels were significantly higher for both sexes after challenge than prior to challenge, returning to baseline levels 5–8 days post-challenge. Wilcoxon signed rank test indicated that the ACTH challenge resulted in significant increase in hormone metabolite levels for corticosterone (*z* = −2.896, *P* = 0.004), T3 (*z* = −3.309, *P* = 0.001), T4 (*z* = −3, *P* = −0.003) and aldosterone (*z* = −2.76, *P* = 0.006). We found no significant difference in progesterone levels (*z* = −0.73, *P* = 0.62) post-ACTH challenge. All non-GC hormones peaked ±1 day relative to the GC peak in the two males, and on the same day as GC for both females (Fig. 5).

Similarly, both male and female TSH-challenged tigers showed a significant increase in post-challenge fecal T3 and T4 levels, with a latency period of 2 days (Fig. 6). Post-TSH challenge, there was a significant increase in the hormone metabolites for T3 (*z* = −2.52, *P* = 0.01), T4 (*z* = −2.380, *P* = −0.01), corticosterone (*z* = −2.52, *P* = 0.01) and aldosterone (*z* = −1.96, *P* = 0.05). There was no significant increase in progesterone (*z* = −0.73, *P* = 0.62) post-TSH challenge. T3 and T4 peaked concurrently in both the male and female (Fig. 6). However, GC exhibited a small peak corresponding with the thyroid peaks and a markedly larger peak 4–7 days later in both sexes relative to the thyroid peaks. Aldosterone showed the same response as GC in the male post-TSH challenge but had a major peak consistent with the thyroid peak in the female (Fig. 6).

### Gestation and lactation period samples

**Figure 7:**
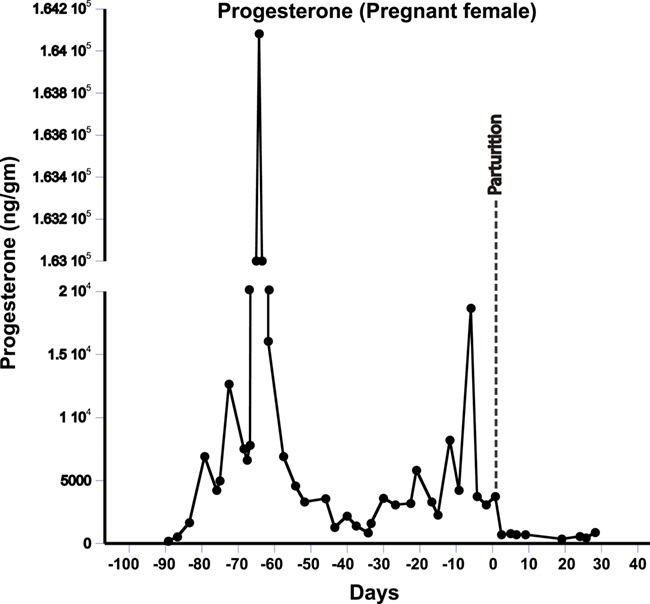
Results of progesterone assays for a pregnant female from conception through early lactation

Mean fecal progesterone, testosterone, A4 and aldosterone levels in the PDZ female were elevated during both gestation and lactation periods. The pregnant female exhibited significantly elevated mean fecal progesterone levels (Fig. 7, Table 2) (4838.49 ± 839.81 ng/g) that were an order of magnitude higher than those of all non-pregnant samples (490.04 ± 51.31 ng/g; *P* < 0.001). This pattern was found to be the same even after removing the few outliers with extreme high progesterone levels (mean 3524.25 ± 40 789 ng/g; *P* < 0.001). Mean testosterone levels was significantly higher in pregnant (162.96 ± 34.86 ng/g; *P* = 0.02) than pre-pregnancy samples (86.67 ± 5.7 ng/g), as was A4 (pregnancy = 2134 ± 317.17; *P* = 0.04 vs. pre-pregnancy = 1501 ± 189.95 ng/g). Mean aldosterone levels were significantly elevated during pregnancy (pregnant = 143094.26 ± 30025.15; *P* = 0.002 vs. non-pregnant = 36 241 ± 3782.13 ng/g).

Progesterone and aldosterone levels were significantly higher in early lactation compared to other non-pregnant states (P4, non-pregnant = 490.04 ± 51.31 ng/g vs. lactating = 1318.39 ± 462.12 ng/g; *P* = 0.004; Aldosterone, non-pregnant = 36 241 ± 3782.13 ng/g vs. lactating = 66818.74 ± 11895.25 ng/g; *P* = 0.03). However, androgens (both T and A4) levels did not show any significant change between both periods (T, non-pregnant = 86.67 ± 5.7 ng/g vs. lactating = 81.06 ± 27.22 ng/g; *P* = 0.42; A4, non-pregnant = 1501 ± 189.95 ng/g vs. lactating = 1353 ± 169.48 ng/g; *P* = 0.25; Table 2). However, all four hormones were significantly lower during lactation compared to the gestation period (Progesterone, gestation = 3524.25 ± 407.89 ng/g vs. lactation = 1318.39 ± 462.12 ng/g; *P* = 0.001; aldosterone, gestation = 143094.26 ± 30025.15 vs. lactation = 66818.74 ± 11895.25 ng/g; *P* = 0.01; testosterone, gestation = 162.96 ± 34.86 ng/g vs. lactation = 86.67 ± 5.7 ng/g; *P* = 0.04; A4, gestation = 2134 ± 317.17 vs. lactation = 1353 ± 169.48 ng/g; *P* = 0.02; Table 2).

## Discussion

Our results show that all the targeted fecal hormone metabolites can be measured reliably and accurately using the assays we investigated. HPLC analysis of GC and thyroid (T3 and T4) hormone assays detected immunoreactive peaks at fractions 71–75, 24 and 32, respectively, consistent with their respective parent hormones (Figs 1 and 2). The testosterone HPLC analyses and subsequent RIA and EIA assays mostly detected metabolites eluting consistent with testosterone, and the A4 RIA assay detected much higher levels of A4 in gestating and lactating females. Most of our HPLC analyses also detected a broad immunoreactive peak in early fractions (fractions 1–20), where more polar steroids are eluted. These peaks likely represent conjugated hormones that cross-react with their assays or free ^3^H cleaved from the added labelled hormone tracer used to standardize elution times. Presence of such polar steroid peaks was found in a number of other studies ranging from herbivores, carnivores and primates (Brown *et al.*, 1994; Velloso *et al.*, 1998; Wasser *et al.*, 2000; Mohle *et al.*, 2002; Dloniak *et al.*, 2004; Putranto 2011). Overall, analysis of the HPLC fragments indicates that the assays measured in this study are able to detect their respective fecal hormone metabolites in tigers, although future studies might also test whether acid solvolysis increases assay reliability by deconjugating any polar metabolites that may have been observed in this study.

ACTH and TSH challenge studies revealed that our GC and thyroid hormone measures reliably reflect adrenal and thyroid biological activity and that the adrenal and thyroid responses to these challenges are not independent in tigers. This is likely also the case in other species (Wasser *et al.*, 2017). All four hormones (GCs, aldosterone, T3 and T4) peaked around the same time in response to ACTH (Fig. 5), although the male response showed more inter-individual variation within and between hormones compared to females. By contrast, the multi-hormone response to TSH showed a several day delay in the peak response of GC (and aldosterone in the male only) relative to the thyroid hormone peaks (Fig. 6). The relatively concurrent responses of adrenal and thyroid hormones to ACTH challenge vs. the delayed adrenal activation relative to thyroid hormone in response to TSH challenge are consistent with actions of these two hormone classes for regulating nutritional and other stresses. GC rapidly mobilizes glucose providing quick energy to respond to emergencies. Concurrent elevation of T3 likely assures that metabolic rate is also high, allowing maximal use of the elevated glucose in healthy individuals. By contrast, thyroid hormone typically operates more slowly, adjusting metabolism as needed, which does not necessarily require concurrent elevation of glucose. Thus, our study suggests that TSH triggers T3 and T4 secretion, elevating metabolic rate, presumably, triggering GC release later, when the elevated metabolic rate depletes glucose. Given the excellent thyroid response to the TSH analog used in this study, we highly recommend its use in future TSH challenge studies.

**Table 2:** Measures of various hormones during different reproductive periods

**Reproductive period**	**Mean hormone levels (ng/g)**			
	**Progesterone (P4)**	**Testosterone (T)**	**Androstenedione (A4)**	**Aldosterone**
**Pre-pregnancy**	490.04 ± 51.31	86.67 ± 5.7	1501 ± 189.95	36 241 ± 3782.13
**Entire pregnancy**	4838.49 ± 839.81	162.96 ± 34.86	2134 ± 317.17	143094.26 ± 30025.15
**Lactation**	1318.39 ± 462.12	81.06 ± 27.22	1353 ± 169.48	66818.74 ± 11895.25

Our analysis of steroid and mineralocorticoid hormone metabolites during gestation and lactation period demonstrates their use in assessing reproductive status in tigers. Results showed that mean concentrations of progesterone, testosterone, A4 and aldosterone were significantly higher during gestation and lactation compared to the non-pregnant state in the same female, as well as in the non-pregnant female (Table 2, Supplementary Fig. S10). However, concentrations of these hormones were also significantly higher during gestation compared to early lactation. Similar patterns of reproductive hormones have been reported in other mammals, including several felids (Brown *et al.*, 1994; Tucker, 2000; Rolland *et al.*, 2005).

Increased levels of aldosterone during pregnancy and lactation is also expected as aldosterone is known to have been associated with stress management (Houser *et al.*, 2011), electrolyte management (Weeks and Kirkpatrick, 1976) and plays an important role in preeclampsia during human pregnancy (Shojaati *et al.*, 2004; Escher and Mohaupt, 2007). Given its importance in environmental physiology (Weeks and Kirkpatrick, 1976; Ortiz *et al.* 2006), aldosterone measurement could have great utility in wildlife health monitoring studies.

Finally, reproductive-state dependent changes in reproductive hormones among the tigers support their application as an index of reproductive status in this species. All the reproductive hormones show similar patterns of elevation, though at different scales, during pregnancy and lactation periods. Hormone ratio profiles (e.g. progesterone: testosterone or progesterone: A4) might be used to distinguish pregnant and early lactation from other non-pregnant states in wild populations. Although the testosterone and A4 profiles are virtually identical during pregnancy, levels of A4 were consistently much higher than testosterone. Similar progesterone: androgen ratios have been used for gender and pregnancy identification (Dloniak *et al.*, 2004; Rolland *et al.*, 2005; Ayers *et al.*, 2012, Wasser *et al.*, 2017), as well as for distinguishing reproductive state and pseudo-pregnancy in wild animals (van Kesteren *et al.*, 2013).

In conclusion, our results suggest that the targeted fecal hormone assays examined in this study can provide reliable indices of the physiological response to environmental stressors and their reproductive consequences among wild populations. Specifically, the validated hormone measures could potentially provide relatively immediate physiological indices of disturbance and/or nutritional stress, as well as the reproductive response to major disturbances including habitat destruction, fragmentation, hunting, tourism and human–animal conflict among tigers, bridging the gaps between environmental events and their demographic outcomes on population growth. Such information could, in turn, highlight what pressures are most in need of mitigation to improve population health assessment and curb population declines.

## Supplementary Material

11_Mondol_et_al_2019_Supplementary_Figures_coz091
