# Supplementary figures and images for: Fecal stress, nutrition and reproductive hormones for monitoring environmental impacts on tigers (Panthera tigris)
Source: Conserv Physiol. 2020 Jan 12;8(1):coz091. doi: 10.1093/conphys/coz091 (PMC6955020; doi:10.1093/conphys/coz091)

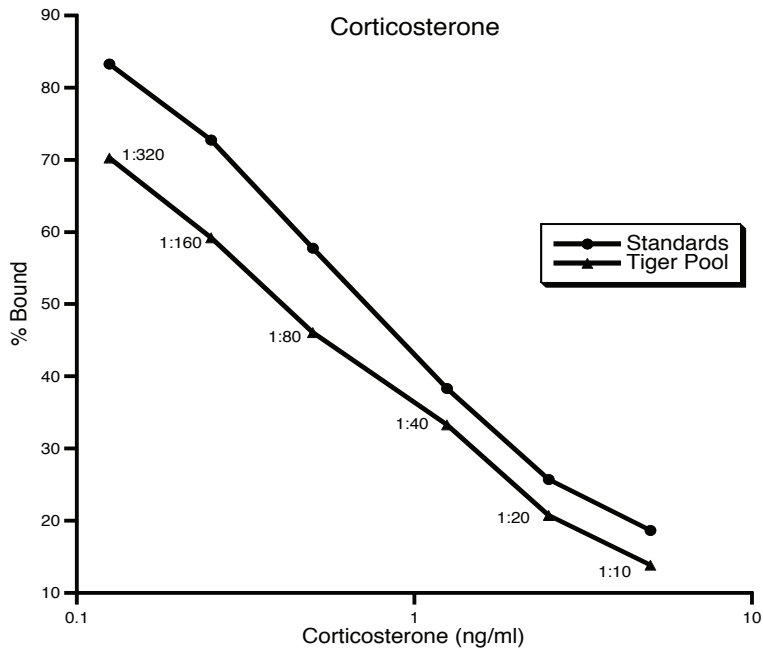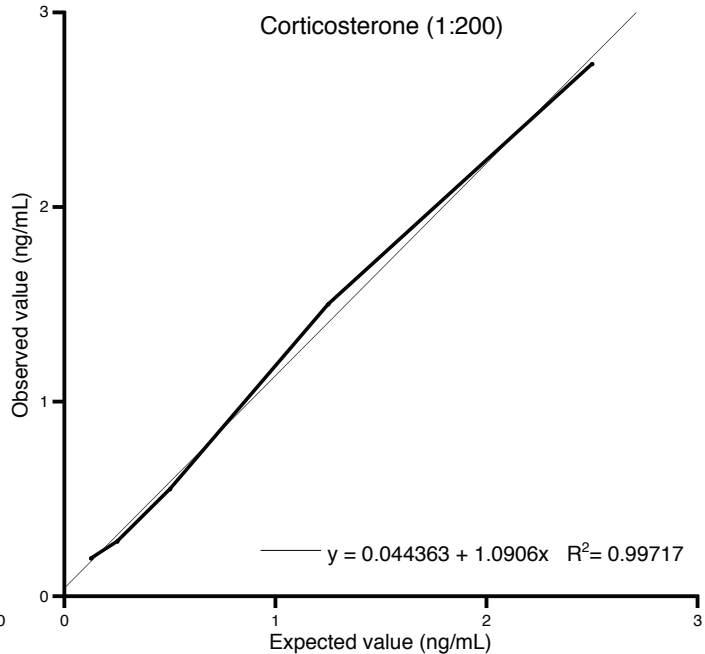

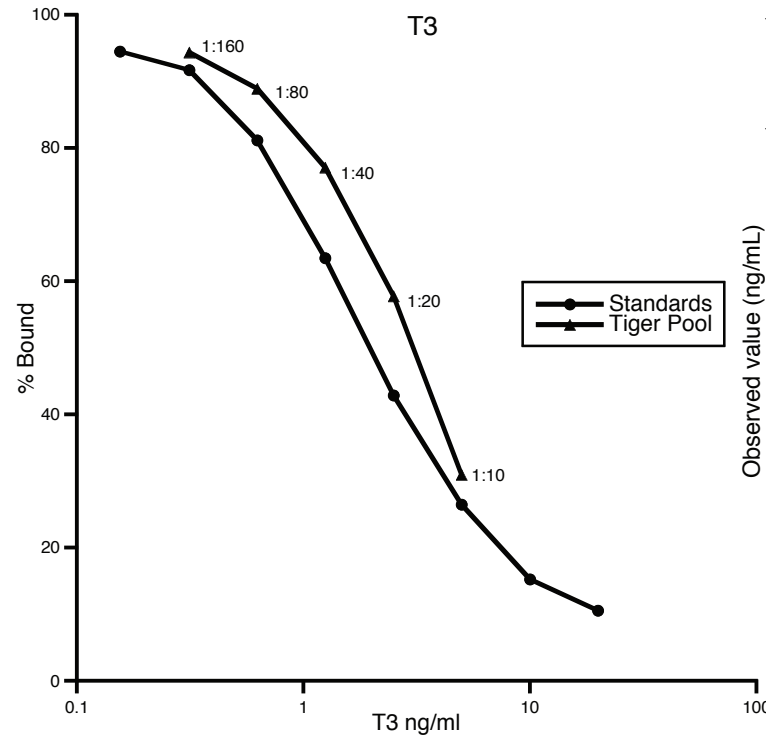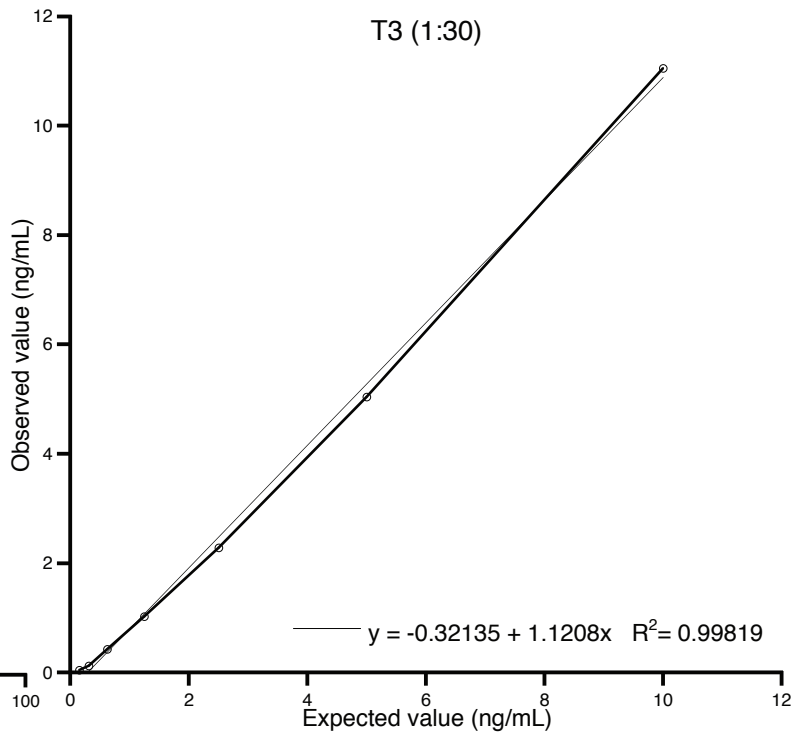

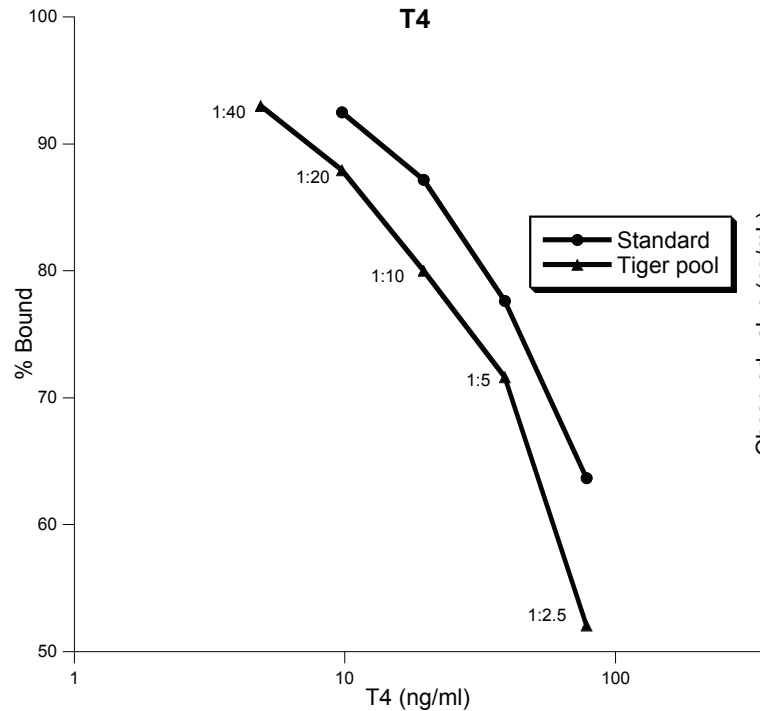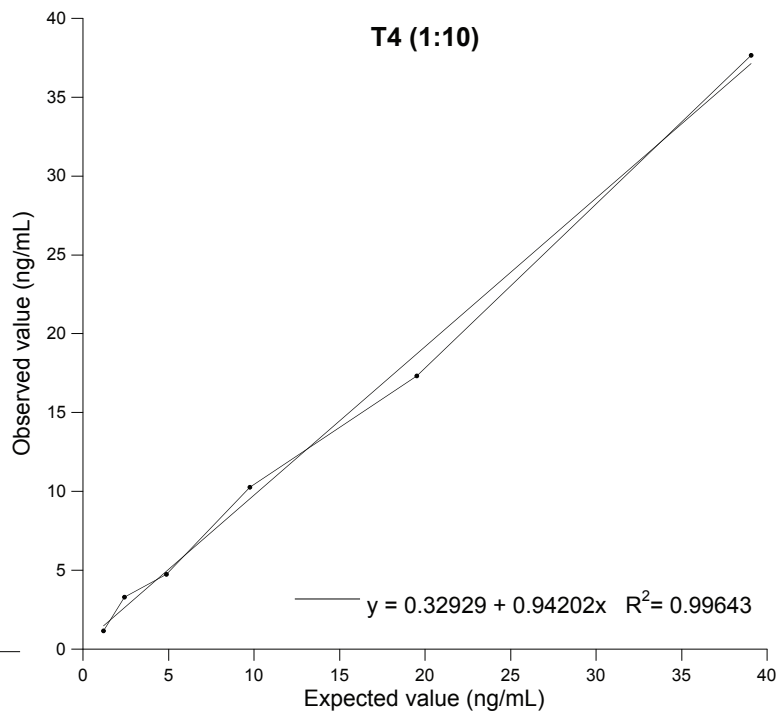

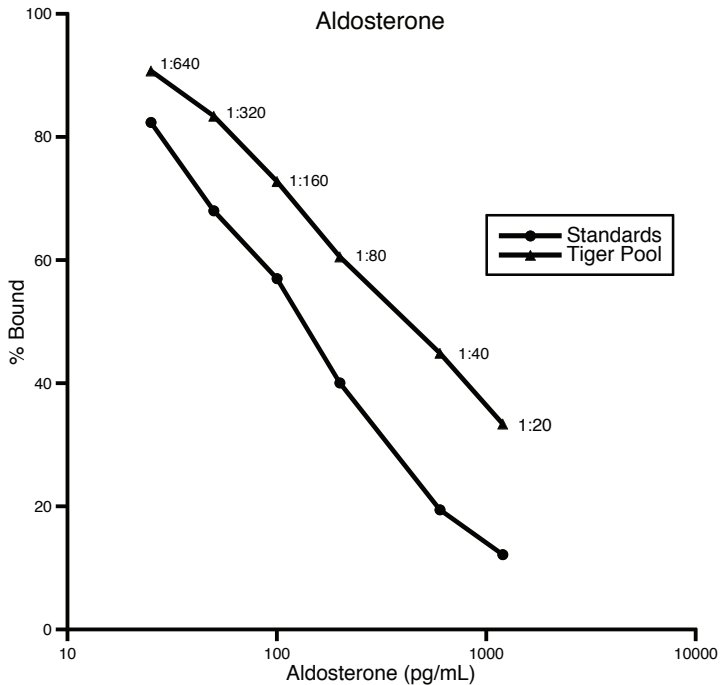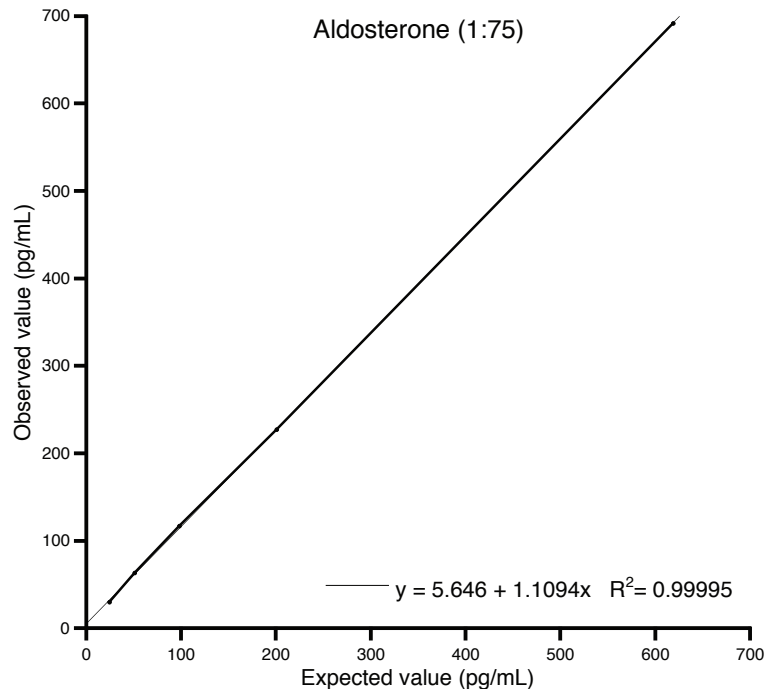

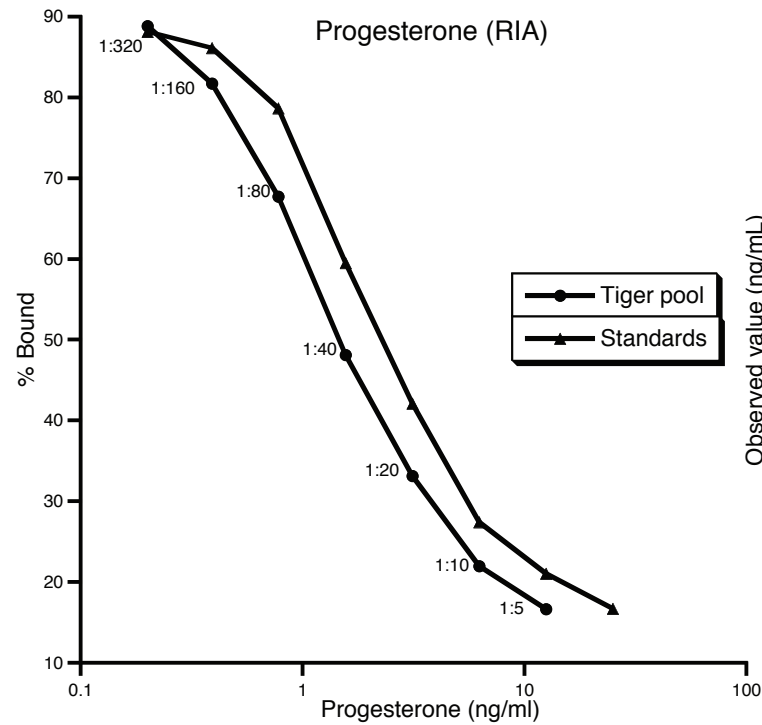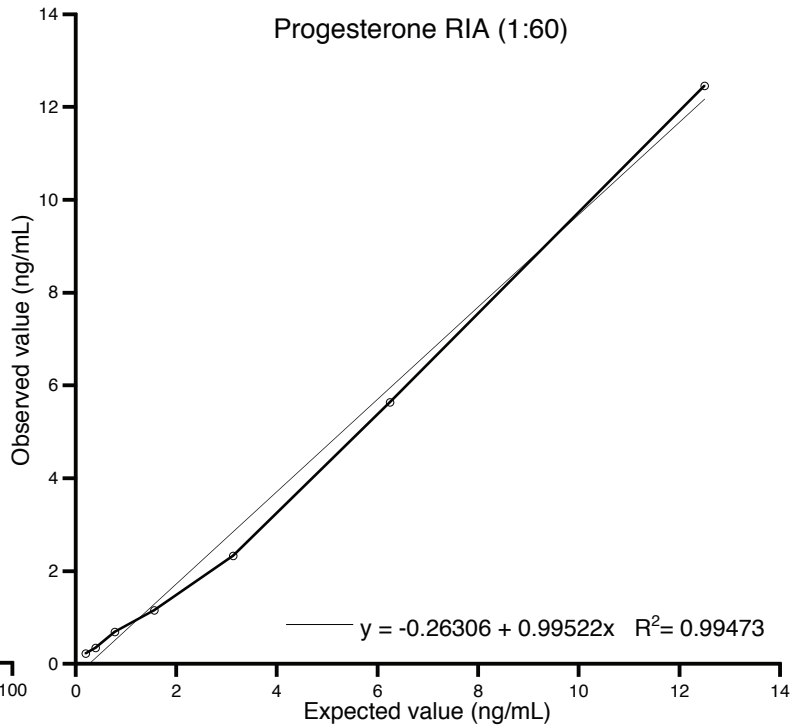

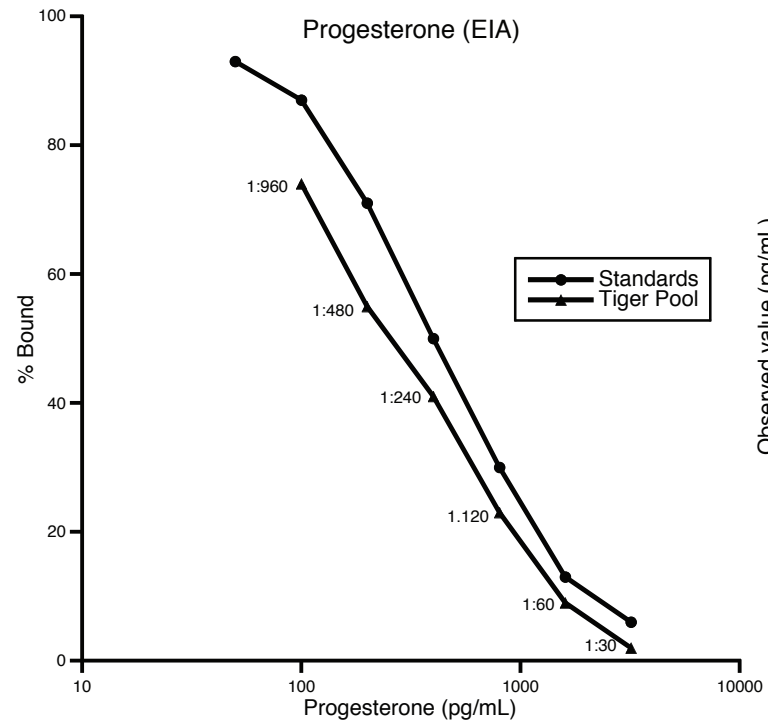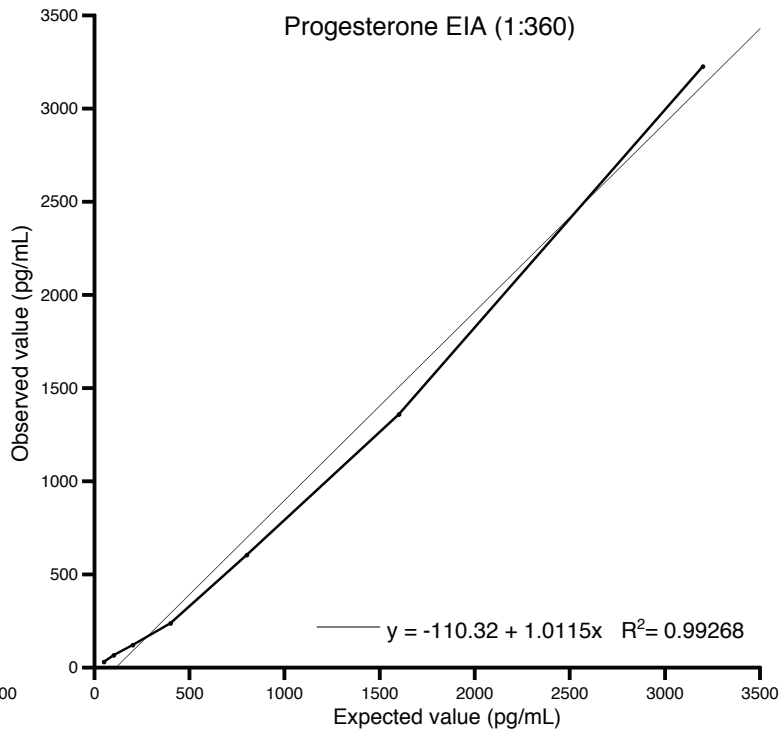

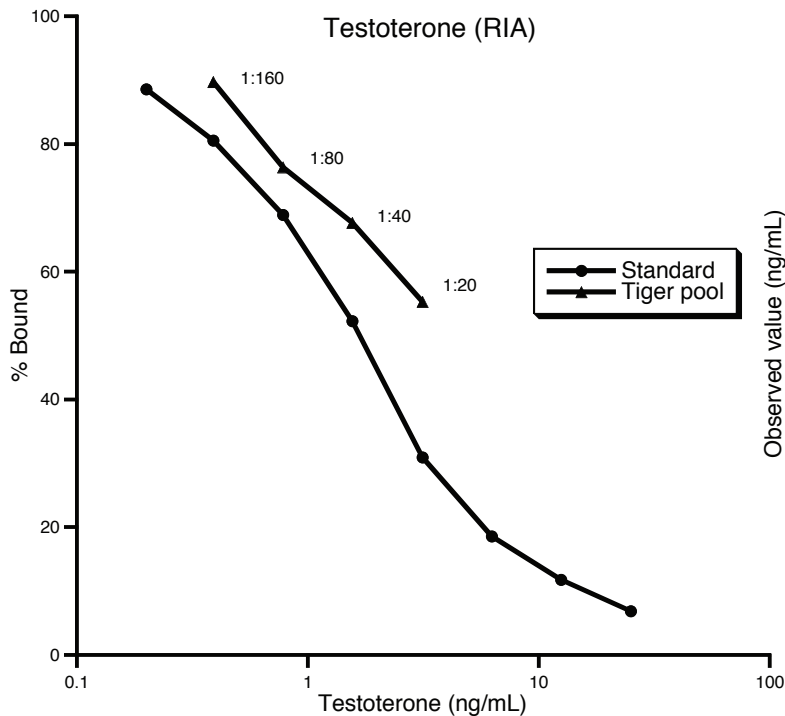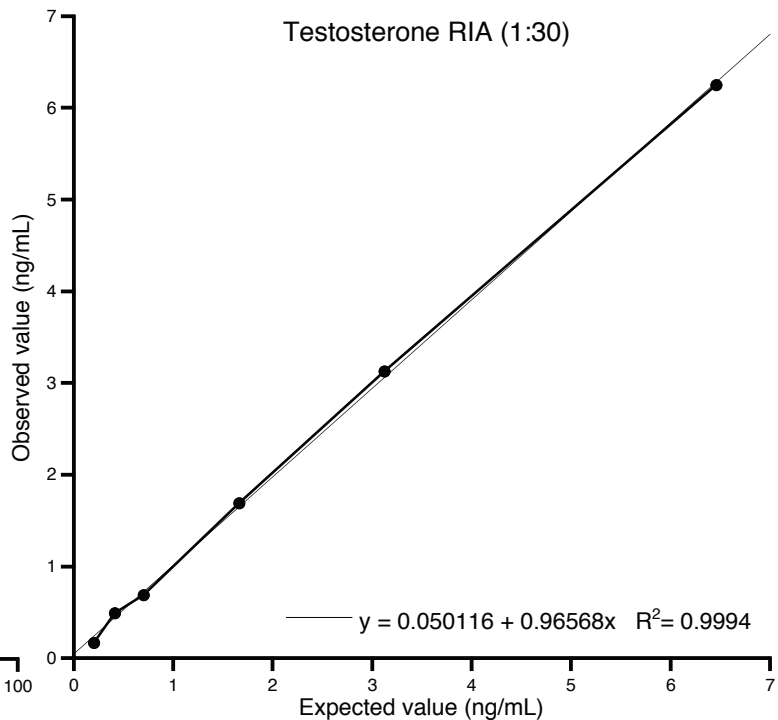

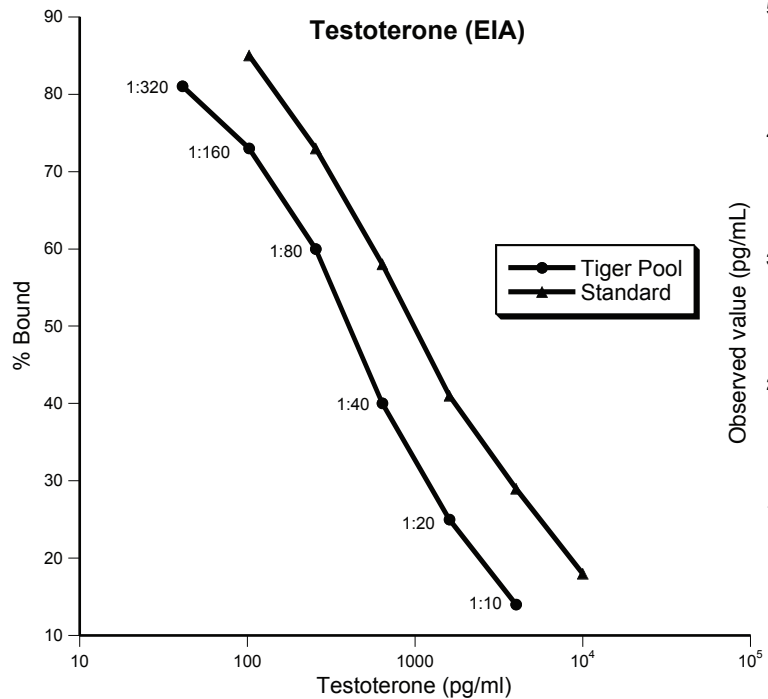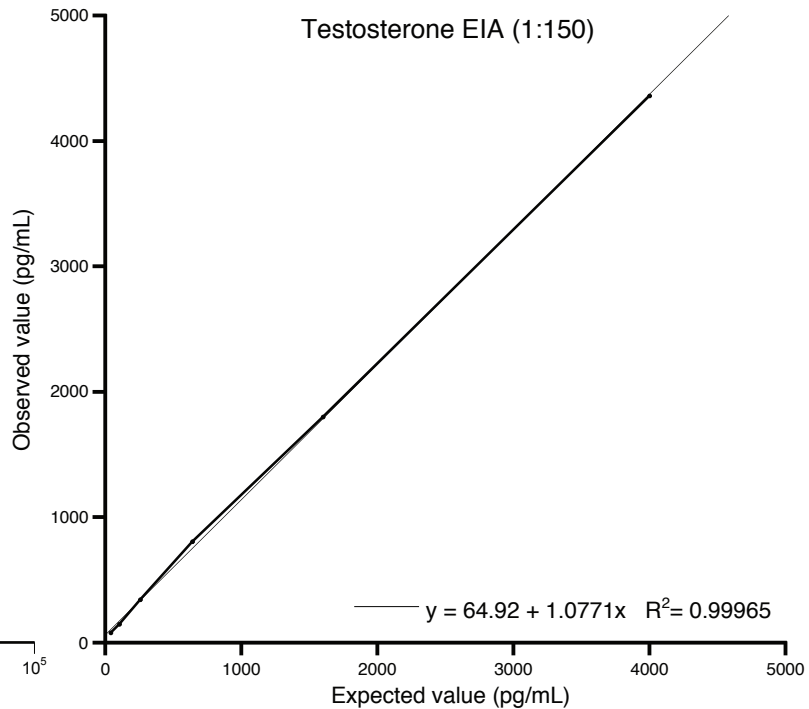

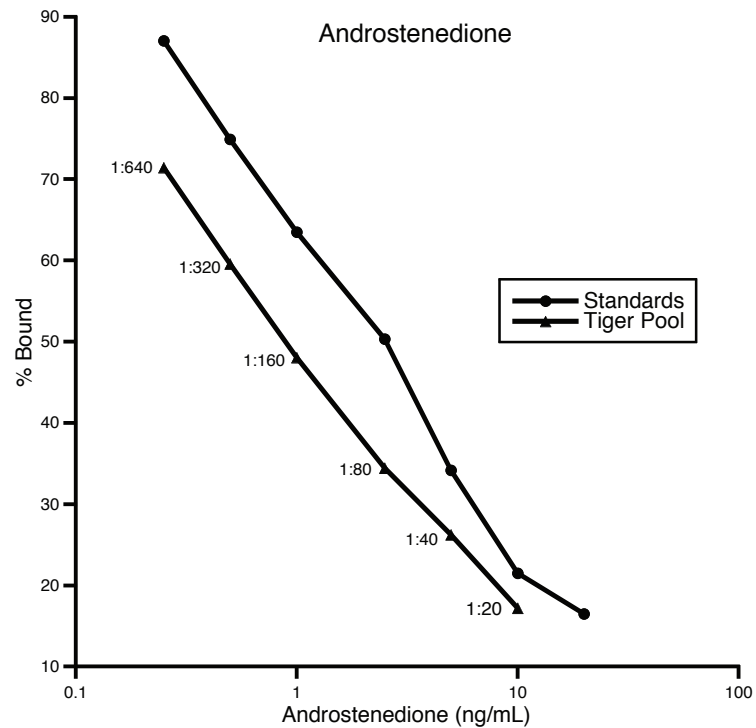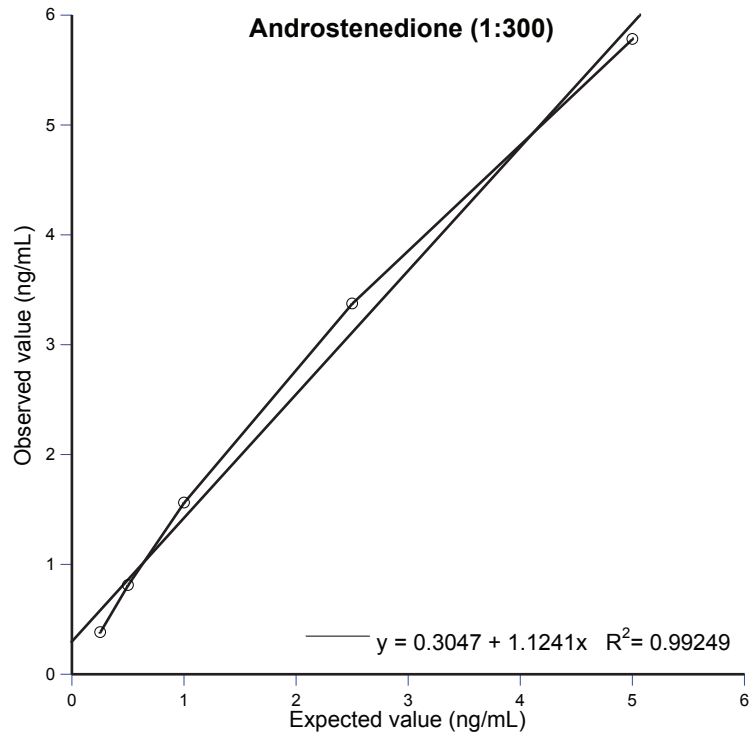

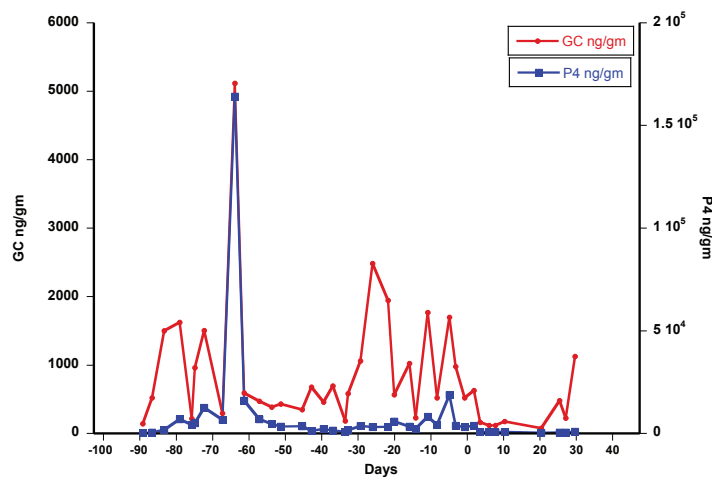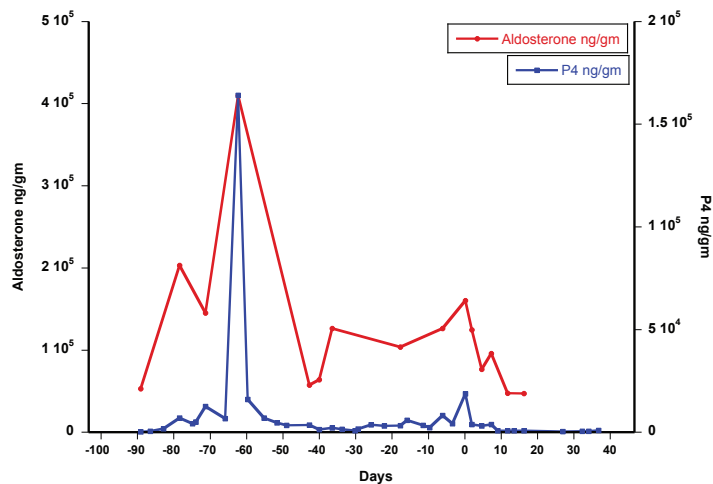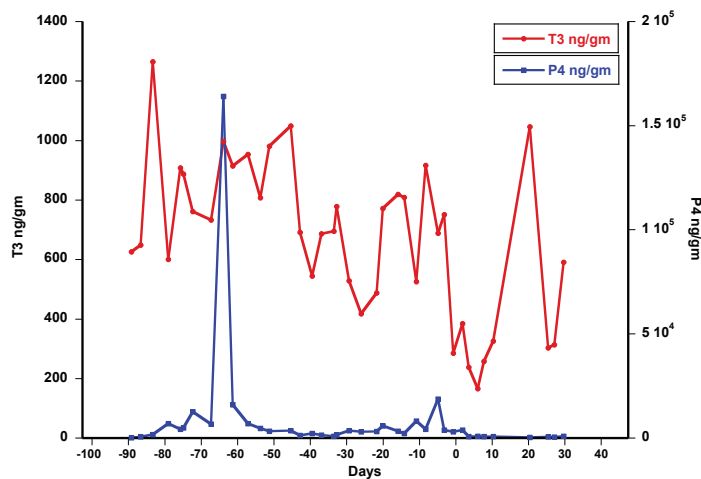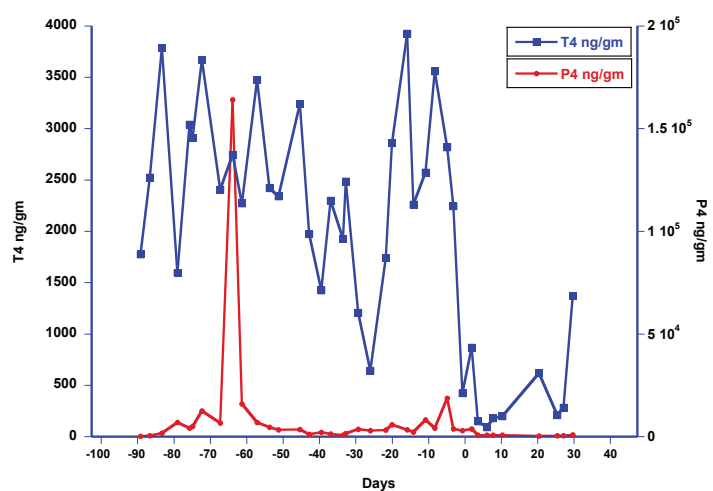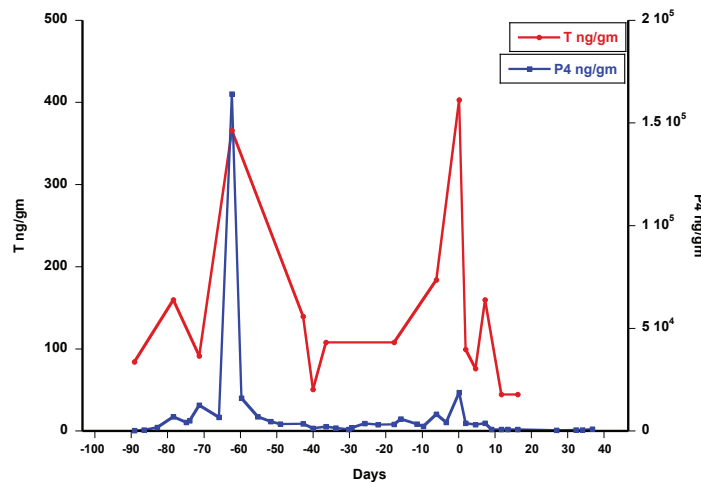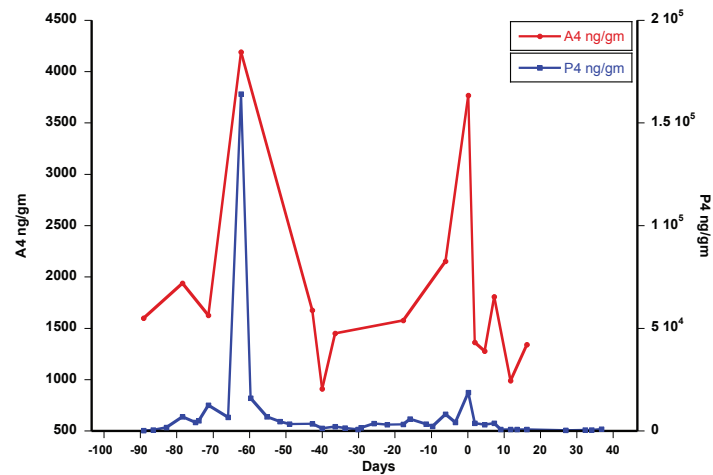

Supplement: 11_Mondol_et_al_2019_Supplementary_Figures_coz091 [file 11_mondol_et_al_2019_supplementary_figures_coz091.pdf]
